# Microbial agents enhance the yield and quality of pears by regulating the composition and networks of microbial communities in the phyllosphere and rhizosphere

**DOI:** 10.3389/fmicb.2026.1763579

**Published:** 2026-02-03

**Authors:** Na Luo, Yulou Zhang, Zhifeng Ren, Xinfeng Wang, Hongbo Li, Aiping Zhang

**Affiliations:** 1 Institute of Environment and Sustainable Development in Agriculture, Chinese Academy of Agricultural Sciences, Beijing, China; 2 Jinhejianong Biotechnology Co., Ltd., Beijing, China

**Keywords:** bacterial communities, fruit quality, microbial agents, phyllosphere, rhizosphere

## Abstract

Microbial management offers a sustainable pathway to enhance crop performance by optimizing plant-associated microbiomes. However, integrated strategies that concurrently target both the rhizosphere and phyllosphere to improve fruit tree productivity and quality remain underexplored. This study systematically evaluated the effects of combined soil and foliar microbial applications on the yield, fruit quality, and microbiome dynamics of ‘Yuluxiang’ pear. We compared conventional fertilization (CK) with two treatments: CK plus a soil-applied anti-replant disease agent (CF) and CK plus both the soil agent and a foliar growth-promoting inoculant (CFW). Microbial applications significantly increased the yield by up to 60.4% in CF treatment, and enhanced key fruit quality parameters, including soluble solids content (increased by 17.2% in CF and 16.7% in CFW) and fruit shape index. These agronomic improvements were closely associated with a targeted restructuring of bacterial communities in both the phyllosphere and rhizosphere. Specifically, beneficial phyla such as Actinomycetota were enriched in the phyllosphere under CFW treatment, while Bacillota increased in the rhizosphere under microbial amendments. Furthermore, co-occurrence network analysis revealed that microbial applications fostered more complex and cooperative microbial networks, with increased nodes and edges across both compartments. This work demonstrates that an integrated soil and foliar microbiome management strategy can mitigate replant disease constraints and elevate fruit quality, providing a practical approach for sustainable orchard production.

## 1 Introduction

Pears, as a key economic fruit within the Rosaceae family, are cultivated on a global scale (Kim et al., 2024). The ‘Yuluxiang’ pear, a prominent cultivar in Chinese orchards, is highly appreciated for its desirable sensory qualities, including a crisp bite, abundant juiciness, and high sweetness (Lozano et al., 2023). However, the reliable expression of these superior traits remains a challenge (Kumar et al., 2019). In many production areas, common issues include undersized fruit, poor skin coloration, and low soluble sugar content, which collectively diminish fruit marketability and grower profitability (Abeyrathna et al., 2023). Refining nutrient management practices is therefore essential to mitigate these constraints and achieve consistent fruit quality.

Conventional chemical fertilization, the traditional orchard management practice, focuses on supplementing macronutrients but fails to address the underlying microbial imbalance and pathogen accumulation in replanted soils. This long-term reliance can exacerbate soil degradation and negatively alter the structure and function of soil microbial communities (Jin et al., 2022). Meanwhile, the sustainable production is critically constrained by replant disease (RD), a pervasive problem in major fruit-producing regions. This issue is exemplified by apple replant disease (ARD), a specific manifestation of RD primarily driven by pathogen accumulation in the rhizosphere, notably fungi such as *Fusarium, Rhizoctonia*, and *Cylindrocarpon* species, and oomycetes like *Pythium* and *Phytophthora* (Geng et al., 2022). A key aggravating factor is the root-secreted toxic accumulation of phenolic acids (e.g., cinnamic acid, phlorizin), which impairs the root antioxidant system and further promotes pathogen proliferation (Duan et al., 2025). These pathological and biochemical stresses collectively result in reduced tree vigor, significant yield losses (20–50%), and deteriorated fruit quality, including lower soluble solid content and firmness (Duan et al., 2022). Conventional control has long relied on broad-spectrum soil chemical fumigants like methyl bromide. However, due to their high toxicity, ozone-depleting potential, and adverse environmental impacts, the search for sustainable alternatives is imperative (Geng et al., 2022). Among these alternatives, soil-applied microbial agents that suppress replant disease offer a promising strategy to mitigate disease pressure and improve plant health (Durán et al., 2021). Furthermore, combining disease-suppressing with growth-promoting microbial agents may yield synergistic benefits, enhancing plant stress resistance and fruit quality through the targeted modulation of soil microbial communities (Mishu et al., 2025). Complementing soil-based strategies, foliar fertilization serves as a targeted nutritional supplement that enhances nutrient use efficiency and directly influences fruit development and metabolism. It may also secondarily alter the composition and interactions of the rhizosphere bacterial community (Demir et al., 2024). Given the vital role of microbial assemblages in nutrient cycling and plant health, this study investigates the effects of microbial agents on the yield, fruit quality, and associated bacterial community dynamics of ‘Yuluxiang’ pears. The objective is to establish a foundation for a more sustainable and effective integrated nutrient management system.

Biological control agents (BCAs), particularly those based on *Bacillus* spp., represent promising eco-friendly alternatives (Duan et al., 2025; Nzimande et al., 2025). Their efficacy stems from multiple mechanisms, including: (1) the synthesis of antimicrobial compounds (e.g., lipopeptides, siderophores, lytic enzymes) and volatile organic compounds (VOCs) for pathogen inhibition; (2) the degradation of phytotoxic phenolic acids; and (3) the modulation of soil microbial communities and key enzyme activities (e.g., urease, phosphatase) to enhance nutrient cycling (Chen et al., 2020; Duan et al., 2022). Field trials with specific strains, such as *Bacillus amyloliquefaciens* QSB-6, have validated these mechanisms, demonstrating significant plant growth promotion alongside reduced pathogen abundance and phenolic acid content in replanted soils (Duan et al., 2022). However, despite this demonstrated potential, current research and practical application remain predominantly focused on the single soil application of these disease-suppressing agents.

This soil-centric focus overlooks the potential of complementary aboveground strategies. The phyllosphere (leaf surface) constitutes a critical, yet distinct, microbial habitat integral to plant health and productivity (Leveau, 2019). The foliar application of plant growth-promoting bacteria (PGPB) can directly colonize the phyllosphere, enhancing photosynthetic efficiency, boosting antioxidant enzyme activities, and improving nutrient absorption (Hirata et al., 2025). This approach has proven effective in various crops. For instance, in sweet cherry, foliar-applied PGPB significantly increased yield, shoot growth, and leaf mineral content (e.g., N, P, K, Fe, Zn) (Esitken et al., 2006). Similarly, in apple, foliar application of *Pseudomonas* and *Bacillus* strains at key phenological stages enhanced plant growth and yield (Kordatzaki et al., 2022). These benefits are mediated through the diverse and complementary mechanisms of action employed by PGPB, which collectively support plant development and stress resilience (Acuña et al., 2024). These can be categorized as direct or indirect. Direct mechanisms enhance plant nutrition and growth, including biological nitrogen fixation, phytohormone production, and mineral solubilization (e.g., phosphorus) (Agake et al., 2022). Indirect mechanisms primarily involve biocontrol, such as synthesizing antibiotics, lytic enzymes, and antifungal metabolites to suppress phytopathogens, and modulating plant defense signaling pathways (Guardiola-Márquez et al., 2022). Collectively, these interactions enhance the plant’s ability to mitigate abiotic and biotic stresses, thereby supporting sustained health and productivity (Efthimiadou et al., 2020). Furthermore, the efficacy of such treatments can be optimized by using indigenous bacterial strains, which are often more competitive and effective due to their adaptation to local conditions (Katsenios et al., 2022).

Microbial co-occurrence networks are a powerful framework for elucidating complex interactions within soil microbiomes (Lin et al., 2024). By identifying statistically robust co-occurrence patterns among microbial taxa, these networks capture both the structural organization and functional potential of microbial communities (Feng et al., 2025). Evidence suggests that more complex and modular networks support enhanced ecosystem functions, including nutrient cycling, organic matter turnover, pathogen suppression, and ultimately, plant productivity (Zhang et al., 2025). For example, introducing rhizobia has been shown to increase fungal network connectivity in the soybean rhizosphere (Xu et al., 2020), while inoculation with *Rhizopus irregularis* can enhance bacterial network complexity in maize roots (Darriaut et al., 2025). Integrating studies of plant phyllosphere and rhizosphere microbiomes with symbiotic network analysis can provide mechanistic insights into microbial community assembly and interactions. However, integrated strategies combining soil-applied disease suppressants with foliar growth promoters remain largely unexplored. This represents both a significant knowledge gap and a promising avenue for developing integrated orchard management systems.

Therefore, to address critical knowledge gaps in the literature, including the lack of comparative application studies, unclear effects on soil and leaf microbial networks, and limited data linking microbial modulation to fruit quality, this study evaluated three management regimes: (1) conventional fertilization (CK); (2) conventional fertilization plus a soil-applied anti-replant disease agent (CF); and (3) conventional fertilization plus the soil-applied agent and a foliar growth-promoting microbial agent (CFW). By analyzing fruit quality, yield, and bacterial community dynamics in the rhizosphere and phyllosphere, this research aims to establish a scientific foundation for an integrated microbiome management strategy to mitigate replant disease and enhance the sustainable production of ‘Yuluxiang’ pears.

## 2 Materials and methods

### 2.1 Experimental site and design

The study was conducted in a high-density orchard located in Xi County, Shanxi Province, China (110°55′E, 36°41′N), a major apple-producing region. The site has a temperate continental monsoon climate and a loess hilly landform. The climate is characterized by hot, rainy summers (average temperature 21 °C; mean rainfall 303 mm) and cold, dry winters (average temperature 3.5 °C; mean rainfall 75.6 mm). The average annual sunshine duration is 2,740.9 h, and the frost-free period is 150–160 days.

The orchard was established in 2015 on flat terrain with deep, fertile soil characterized by moderately brown topsoil. The experimental tree species is the 11-year-old ‘Yuluxiang’ pear (Pyrus bretschneideri), planted in an east–west orientation with a spacing of 1.0 meters × 3.5 meters. The experiment included three treatments: conventional fertilization control (CK), conventional fertilization plus anti-replant disease agent (CF), and conventional fertilization plus anti-replant disease agent combined with plant growth-promoting microbial inoculant (CFW). A randomized block design was employed, with five replicates per treatment, establishing a total of 15 experimental plots.

The control treatment (CK) followed conventional local fertilization practices, which consisted of applying 2 tons of organic fertilizer per mu (approximately 0.067 hectares) in autumn via 30 cm deep strip trenches placed on one side of each tree. For the CF treatment, 4 kg per mu of a commercial anti-replant disease agent was incorporated into this standard fertilizer regimen. This agent is a microbial consortium primarily composed of *Bacillus amyloliquefaciens*, *Bacillus subtilis*, *Trichoderma harzianum*, *Bacillus pumilus*, *Paenibacillus mucilaginosus*, and *Clonostachys* spp., with a guaranteed minimum viable count of ≥1.0 × 10^9^ CFU mL^−1^. The CFW treatment received the same soil application of the anti-replant disease agent, supplemented with a foliar spray of a growth-promoting microbial inoculant. The foliar inoculant, with a viable count of ≥1.0 × 10^9^ CFU mL^−1^, contained a consortium of *Bacillus amyloliquefaciens*, *Bacillus subtilis*, and *Trichoderma harzianum*. The foliar application was performed twice: during the fruit expansion stage (mid-July) and the fruit maturation stage (early August). All fertilizers and microbial amendments were supplied by Jinhua Jianong (Beijing) Biotechnology Co., Ltd.

### 2.2 Sample collection

During the pear tree’s late expansion and ripening stages, leaf samples and rhizosphere soil samples were collected one week after spraying the microbial foliar agent. Five pear trees were randomly selected from each experimental plot. Ten healthy, fully expanded leaves were randomly collected from each tree, covering the upper, middle, and lower canopy layers and distributed across the four cardinal directions (east, west, south, north). Collected leaves were immediately placed in sterile plastic bags. Beneath each pear tree, roots were gently excavated with a shovel. Loose soil adhering to the roots was shaken off, while tightly adhering rhizosphere soil was brushed off and collected. This rhizosphere soil was sieved through a 2 mm mesh and placed into sterile bags. Analyze each experimental plot as an independent biological replicate, consolidating multiple subsamples collected from the same plot. Leaf and soil samples were stored at −80 °C for subsequent DNA isolation and characterization of leaf surface and rhizosphere bacterial communities.

### 2.3 Measurement of yield and quality indicators

In each plot, one pear tree is randomly selected. Using an electronic balance with an accuracy of 0.1 kilograms, the fruit yield of each pear tree is measured to determine the total yield of the entire tree. The yield per unit area was extrapolated based on the orchard planting density using the formula:


$$\begin{array}{c} \text{Yield}\;\mathrm{per}\;\text{unit area}\;(\mathrm{kg}\;{\mathrm{ha}}^{-1})=\text{Yield}\;\mathrm{per}\;\text{plant}\;(\mathrm{kg}\;{\text{plant}}^{-1})\\ \times \text{Planting density}\;(\text{plants}\;{\mathrm{ha}}^{-1}) \end{array}$$

The planting density was calculated from the specific row (3.5 m) and plant (1.0 m) spacing.

From each cluster, randomly select 10 fruits and measure the maximum vertical height (from the base of the receptacle to the apex of the calyx) and the maximum equatorial diameter using a DL91150 electronic digital caliper. These parameters are recorded as longitudinal diameter and transverse diameter, respectively, with an accuracy of 1 millimeter. The fruit shape index was calculated as the ratio of longitudinal diameter to transverse diameter. Subsequently, pulp tissue was uniformly collected along the fruit’s equatorial region, pooled, and ground in a mortar. The ground mixture was filtered through four layers of sterile gauze to obtain clear juice. Total water-soluble solids content in the juice was determined using a handheld refractometer.

From each plot, 10 additional randomly selected fruits exhibiting uniform size, minimal mechanical damage, and no insect infestation were equilibrated at room temperature (20 ± 1 °C) for at least 2 h. A texture analyzer was then used to perform puncture tests on two relatively smooth areas of the fruit’s equatorial region, measuring fruit firmness.

### 2.4 DNA extraction, PCR, Illumina sequencing, and bioinformatics analysis

DNA was extracted from treated leaf samples and rhizosphere soil samples using the E. Z. N. A.® Plant DNA Kit (Omega Bio-tek, Norcross, GA, U. S.) and the FastDNA® Soil Spin Kit (MP Biomedicals, U. S.), respectively, according to the instructions. The integrity and purity of extracted DNA were assessed by 1% agarose gel electrophoresis, and concentration as well as purity were measured with a NanoDrop® One spectrophotometer (Thermo Fisher Scientific, USA). RCR amplification was performed using the extracted DNA as a template, employing specific bacterial primers 338F and 806R (F: 5′-ACTCCTACGGGAGGCAGCA-3′; R: 5′- GGACTACHVGGGTWTCTAAT-3′). Amplifications were carried out using Premix Taq polymerase (Takara Biotechnology, Dalian, China). PCR products were quantified with GeneTools analysis software (v4.03.05.0, SynGene). Based on concentration, amplicons were pooled in equimolar ratios and purified with the E. Z. N. A.® Gel Extraction Kit (Omega Bio-tek, Norcross, GA, USA). The target DNA fragments were eluted in TE buffer, and the final DNA concentration was determined using a NanoDrop 2000C spectrophotometer (Thermo Fisher Scientific). After equalizing the samples, a paired-end (PE) library was constructed using a NEXTFLEX Rapid DNA-Seq Kit. Equal amounts of purified amplicon were pooled and subjected to high-throughput sequencing (Illumina Nextseq 2000 platform PE150 sequencing mode; Majorbio Bio-pharm Technology, Shanghai, China).

Sequencing data underwent denoising using QIIME 2 (Caporaso Lab, Northern Arizona University, Flagstaff, AZ, USA) combined with the DADA2 plugin (Benjamin Callahan, North Carolina State University, Raleigh, NC, USA) on optimized sequences following quality control assembly. Sequences processed by DADA2 are typically referred to as ASVs (Amplifier Sequence Variants). Sequences annotated to chloroplast and mitochondrial origins were removed from all samples. To minimize the impact of sequencing depth on subsequent alpha and beta diversity analyses, the number of sequences per sample was downsampled to 20,000. After downsampling, the average sequence coverage (Good’s coverage) per sample remained at 99.09%. Species taxonomic analysis of ASVs was performed using the Naive Bayes classifier in Qiime2, based on the Sliva 16S rRNA gene database (v 138.2). In this study, the microbial raw sequences were deposited in the SRA database short-read archive SRR36362282.

### 2.5 Statistical analysis

All statistical analyses were performed using IBM SPSS Statistics (v27.1) and R (v4.3.1). All statistical analyses were conducted with experimental plots as independent biological replicates (*n* = 5 per treatment). Microbial sequencing data were derived from pooled samples across all plots. For agronomic data (yield and fruit quality parameters), treatment effects were assessed by one-way analysis of variance (ANOVA). When ANOVA indicated a significant overall effect (*p* < 0.05), means were compared using Tukey’s honestly significant difference (HSD) post-hoc test. General data visualization was performed using GraphPad Prism (v9.5). Bacterial community analyses were conducted in R. Alpha diversity (Chao1 and Shannon) was analyzed via QIIME2, the ggplot2 package (3.5.1) in R software, and SPSS 27.1. Differences in bacterial community structure (beta-diversity) among treatments were tested using Permutational Multivariate Analysis of Variance (PERMANOVA) based on Bray–Curtis dissimilarities with 9,999 permutations, implemented in the vegan package (v2.6–4). The homogeneity of group dispersions was checked. Principal coordinates analysis (PCoA) based on Bray–Curtis distance was used to visualize community composition.

Microbial co-occurrence networks were constructed separately for each treatment and compartment. Pairwise Spearman correlations were calculated among operational taxonomic units (OTUs) with a prevalence > 10%. Only robust correlations with |r| > 0.7 and a *p*-value < 0.01 were retained. Networks were built and their topological properties (nodes, edges, average degree, modularity) were calculated using the igraph package (v1.5.1). Mantel tests based on Pearson’s correlation were performed between the Bray–Curtis distance matrix of bacterial communities and the Euclidean distance matrices of pear yield and quality traits, and the relative abundance of dominant bacterial phyla.

## 3 Results

### 3.1 Impact of soil and foliar microbial applications on yield and quality of pear

Microbial agent applications significantly enhanced the yield and quality of ‘Yuluxiang’ pears (Table 1). The CF treatment produced the highest yield (37,035 kg ha^−1^), which was significantly greater than both the CFW (33,660 kg ha^−1^) and CK (23,085 kg ha^−1^) treatments. This constitutes a 60.4% yield increase over the conventional fertilization (CK) treatment. Fruit quality was significantly influenced by the microbial treatments. Both microbial treatments significantly enhanced fruit sweetness. The soluble solids content (SSC) reached 15.67% in CF and 15.60% in CFW, representing increases of 17.2 and 16.7%, respectively, over the CK. The fruit shape index indicated that microbial applications resulted in a shape closer to spherical (1.0). The indices for CF (0.94) and CFW (0.96) were both significantly higher than that of CK (0.86). For firmness, the CFW treatment yielded the highest value (4.43), which was significantly greater than that of CF (4.33) and CK (4.10).

**Table 1:** Fruit yield and quality of ‘Yuluxiang’ pears under different treatments.

Treatment	Yield (kg ha^−1^)	Soluble solids content%	Fruit shape index	Fruit firmness (kg cm^−2^)
CK	23,085 ± 184.6 c	13.37 ± 0.34 b	0.86 ± 0.02 b	4.10 ± 0.12 b
CF	37,035 ± 245.9 a	15.67 ± 0.35 a	0.94 ± 0.02 a	4.33 ± 0.15 ab
CFW	33,660 ± 211.6 b	15.60 ± 0.26 a	0.96 ± 0.02 a	4.43 ± 0.09 a

Data are presented as the mean ± standard deviation (SD). Different lowercase letters in the same column indicate significant differences among treatments (*p* < 0.05). CK, conventional fertilization, control; CF, conventional fertilization with the addition of an anti-replant disease agent; CFW, conventional fertilization with the addition of an anti-replant disease agent and foliar application of a growth-promoting microbial agent.

### 3.2 Dynamics of phyllosphere and rhizosphere bacterial communities

Alpha diversity of bacterial communities differed markedly between the phyllosphere and rhizosphere and was significantly affected by treatment (Table 2). In the phyllosphere, and most notably at the fruit maturation stage, treatment effects were pronounced. Regarding richness, the CFW treatment significantly reduced the Chao1 index compared to the CK and CF treatments (*p* < 0.05). Similarly, for community diversity, the Shannon index was lowest under the CFW treatment, showing a significant decrease relative to both CK and CF (*p* < 0.05). In contrast, the CF treatment alone significantly increased the Shannon index compared to the CK control (*p* < 0.05). In the rhizosphere, both microbial treatments (CF and CFW) resulted in moderate reductions in bacterial richness during the fruit expansion stage compared to CK (*p* < 0.05). However, during fruit maturation, the CF treatment substantially enhanced richness relative to CK (*p* < 0.05). In contrast to these shifts in richness, Shannon diversity indices remained relatively consistent across all treatments and both growth stages.

**Table 2:** Alpha diversity of phyllosphere and rhizosphere bacterial communities in ‘Yuluxiang’ pears under different treatments at two growth stages.

Phyllosphere	Fruit expansion stage		Fruit maturation stage	
	Chao	Shannon	Chao	Shannon
CK	57.26 ± 11.85	1.85 ± 0.44	68.50 ± 6.73 a	1.41 ± 0.07
CF	72.85 ± 10.21	2.81 ± 0.22	68.07 ± 13.58 a	1.75 ± 0.26
CFW	61.48 ± 9.03	2.64 ± 0.23	39.40 ± 3.82 b	1.23 ± 0.10

Rhizosphere	Fruit expansion stage		Fruit maturation stage	
	Chao	Shannon	Chao	Shannon
CK	474.10 ± 28.88	4.97 ± 1.39	443.87 ± 18.09 b	4.94 ± 0.03 b
CF	434.22 ± 9.76	4.79 ± 0.04	527.69 ± 12.01 a	5.13 ± 0.06 a
CFW	439.60 ± 35.65	4.77 ± 0.16	495.35 ± 16.63 a	4.98 ± 0.06 b

Data are presented as the mean ± standard deviation (SD). Different lowercase letters in the same column indicate significant differences among treatments (*p* < 0.05). CK, conventional fertilization, control; CF, conventional fertilization with the addition of an anti-replant disease agent; CFW, conventional fertilization with the addition of an anti-replant disease agent and foliar application of a growth-promoting microbial agent.

**Figure 1:**
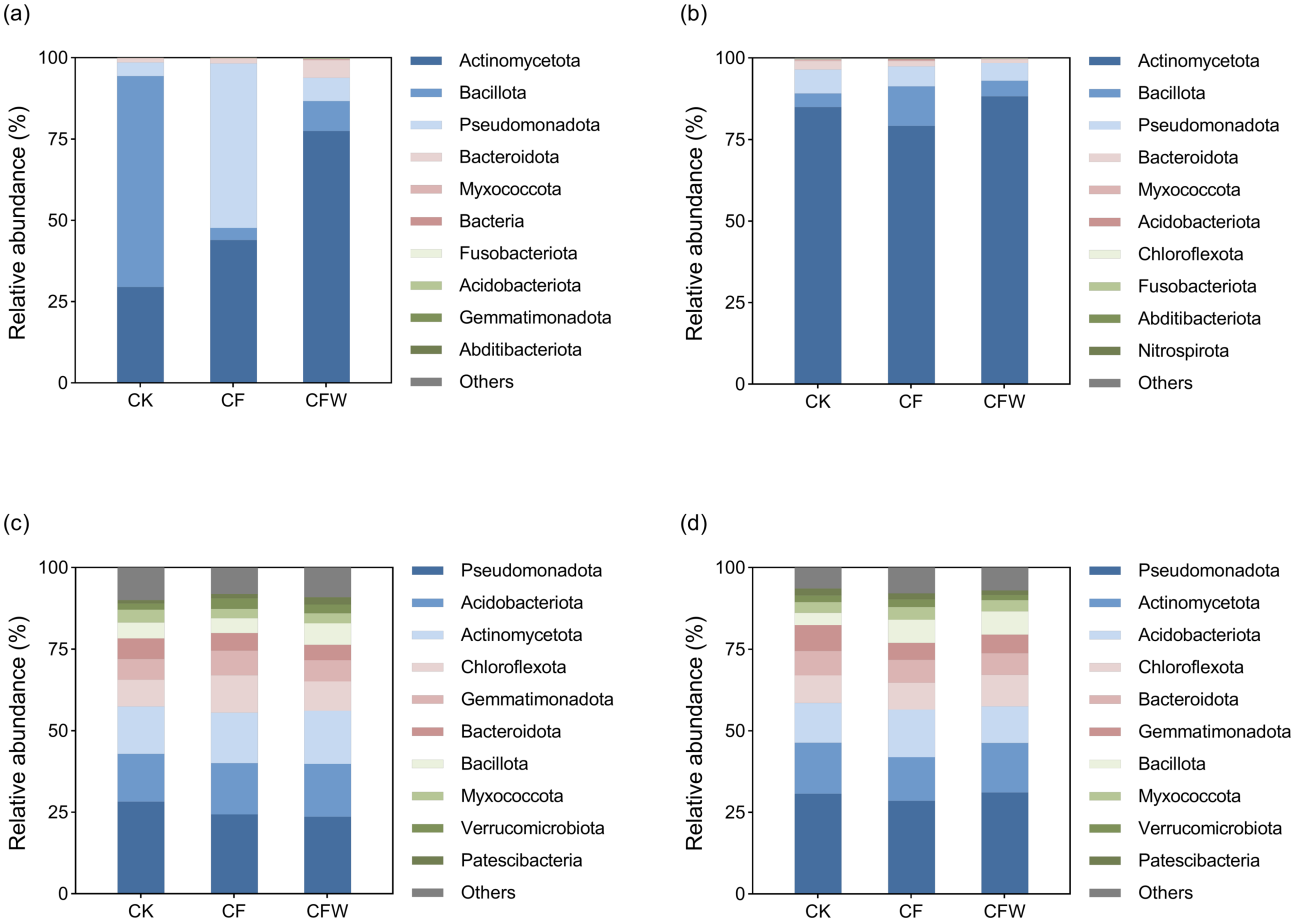
Mean relative abundance of bacteria at the phylum level during the fruit expansion stage and maturation stage in the phyllosphere **(a,b)** and rhizosphere **(c,d)**, respectively. CK, conventional fertilization, control; CF, conventional fertilization with the addition of an anti-replant disease agent; CFW, conventional fertilization with the addition of an anti-replant disease agent and foliar application of a growth-promoting microbial agent.

The application of microbial agents significantly altered bacterial community composition at the phylum level in both the phyllosphere and rhizosphere, with more pronounced shifts occurring in the phyllosphere (Figure 1). During the fruit expansion stage, these phyllosphere communities were restructured. The CF treatment dramatically reduced the abundance of Bacillota from 64.85% (CK) to 3.70%, while promoting Pseudomonadota, which increased from 4.21 to 50.62%. The relative abundance of Actinomycetota also increased from 29.50 to 43.96% under the CF treatment. In contrast, the CFW treatment predominantly enriched Actinomycetota (77.43%), markedly reducing both Bacillota (9.23%) and Pseudomonadota (7.19%) relative to CK. During the fruit maturation stage, Actinomycetota remained the dominant phylum across all treatments, reaching its highest relative abundance (88.19%) in the CFW treatment. Notably, the CF treatment sustained a higher proportion of Bacillota (12.13%) than both CK (4.22%) and CFW (4.80%).

**Figure 2:**
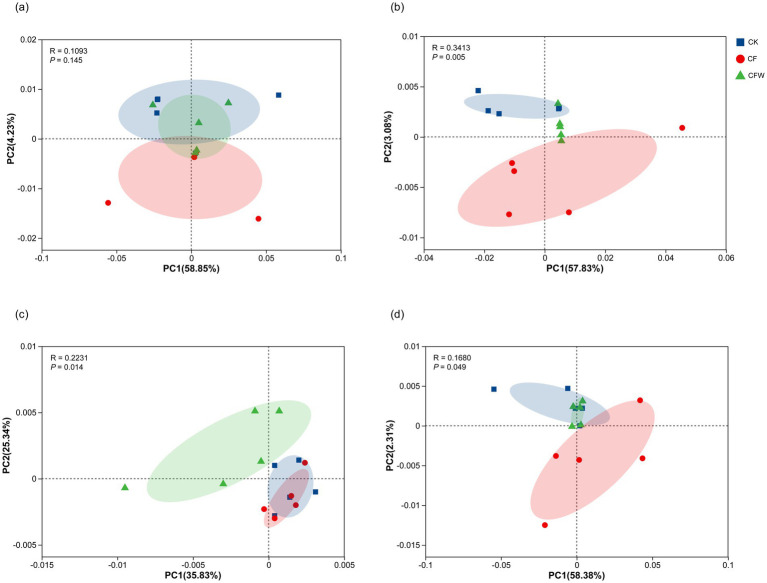
PCoA of bacterial communities based on Bray-Curtis distance in phyllosphere **(a,b)** and rhizosphere **(c,d)** under different treatments at fruit expansion stage and maturation stage. CK, conventional fertilization, control; CF, conventional fertilization with the addition of an anti-replant disease agent; CFW, conventional fertilization with the addition of an anti-replant disease agent and foliar application of a growth-promoting microbial agent.

In contrast to the phyllosphere, the rhizosphere bacterial communities exhibited greater stability in response to the microbial treatments. During the fruit expansion stage, Pseudomonadota remained the dominant phylum across all treatments, with CK, CF, and CFW showing relative abundances of 28.26, 24.35, and 23.56%, respectively. Acidobacteriota and Actinomycetota abundances were stable, although Actinomycetota showed a slight increase from 14.51% (CK) to 16.30% (CFW). Similarly, Chloroflexota and Gemmatimonadota displayed minimal fluctuations among treatments. This pattern of stability persisted into the fruit maturation stage. Pseudomonadota maintained its dominance, and Actinomycetota abundance remained stable, while Acidobacteriota decreased by 7.2% in CFW compared to CK. Notably, Bacillota abundance increased significantly in both CF and CFW treatments compared to CK, representing an 89.1% increase. Principal coordinates analysis (PCoA) based on Bray-Curtis distances revealed distinct clustering of bacterial communities according to treatment (Figure 2). In the phyllosphere during the fruit maturation stage, communities from CFW treatments formed distinct clusters separate from the CK control (PERMANOVA, *p* < 0.05), with PC1 explaining 57.83% of the variance. In contrast, rhizosphere communities demonstrated a different response pattern. During the fruit expansion stage, communities from CFW treatments formed distinct clusters separate from the CK control (PERMANOVA, *p* < 0.05), with PC1 explaining 35.83% of the variance.

### 3.3 Dynamic changes in the network structure of bacterial communities on leaf surfaces and in the rhizosphere

**Figure 3:**
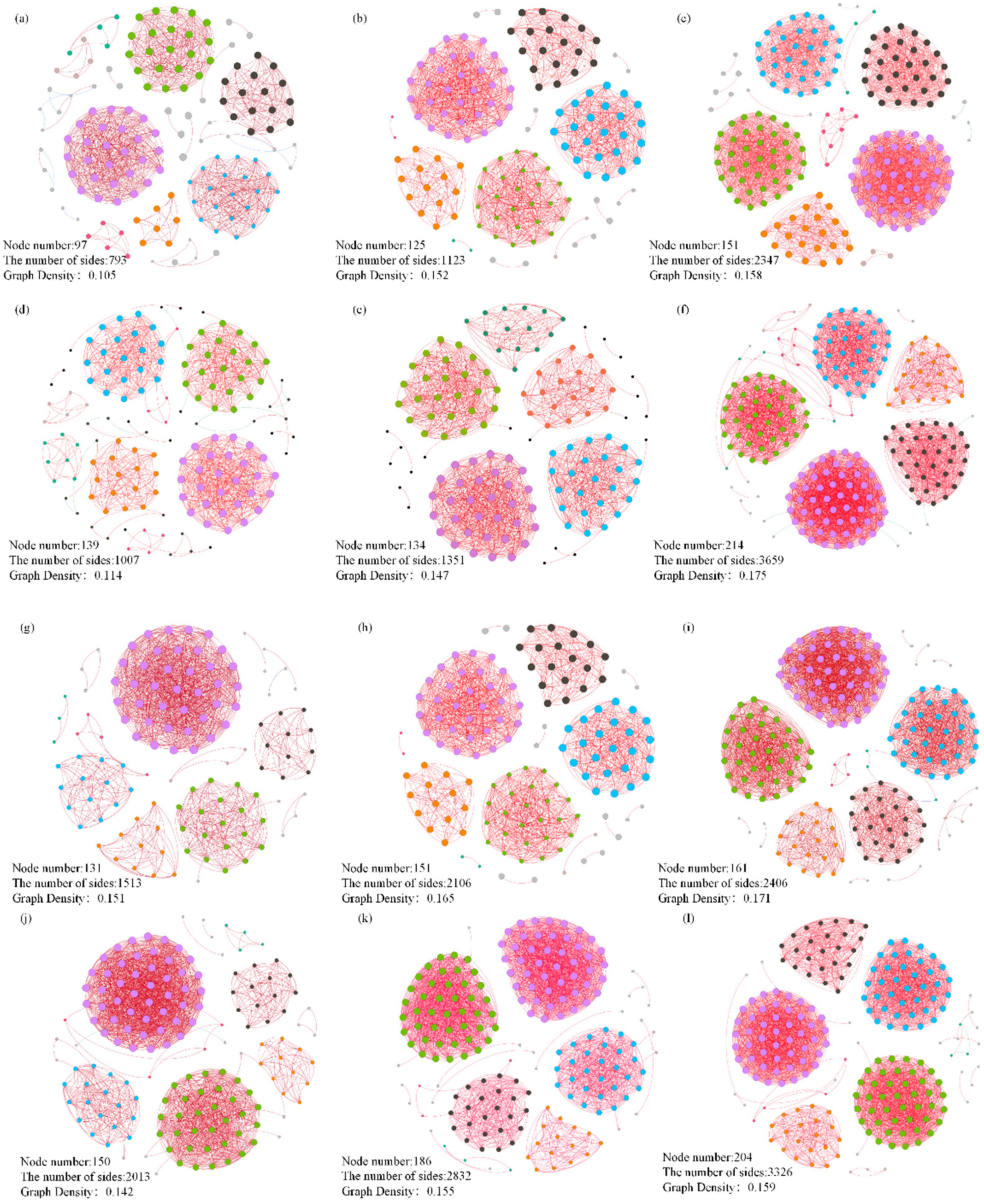
Microbial networks in the phyllosphere **(a–f)** and rhizosphere **(g–l)** regions under different fertilization treatments during the fruit expansion stage **(a–c, g–i)** and maturation stage **(d–f, j–l)**. CK represents conventional fertilization **(a,d,g,j)**; CF denotes conventional fertilization with the addition of an anti-replant disease agent **(b,e,h,k)**; CFW indicates conventional fertilization with the addition of an anti-replant disease agent and foliar application of a growth-promoting microbial agent **(c,f,i,l)**.

Fertilization regimes significantly influenced the topological structure of bacterial co-occurrence networks in both the rhizosphere and phyllosphere of ‘YuluXiang’ pear (Figure 3). Compared to the CK control, microbial amendments (CF and CFW) generally increased network complexity (i.e., the number of nodes and edges) across both sampling periods. During the fruit expansion stage, compared to CK, the CF treatment augmented the network by 28 nodes and 430 edges. The CFW treatment exerted a more substantial influence, increasing the network by 54 nodes and 1,554 edges relative to CK. During the fruit maturation stage, the effect of the CF treatment diminished relative to the expansion stage, with only 5 additional nodes and 344 edges. In contrast, CFW maintained a strong effect, increasing node and edge counts by 75 and 2,652, respectively, compared to CK.

### 3.4 Interrelationships between bacterial community composition and yield, quality, and dominant bacteria

**Figure 4:**
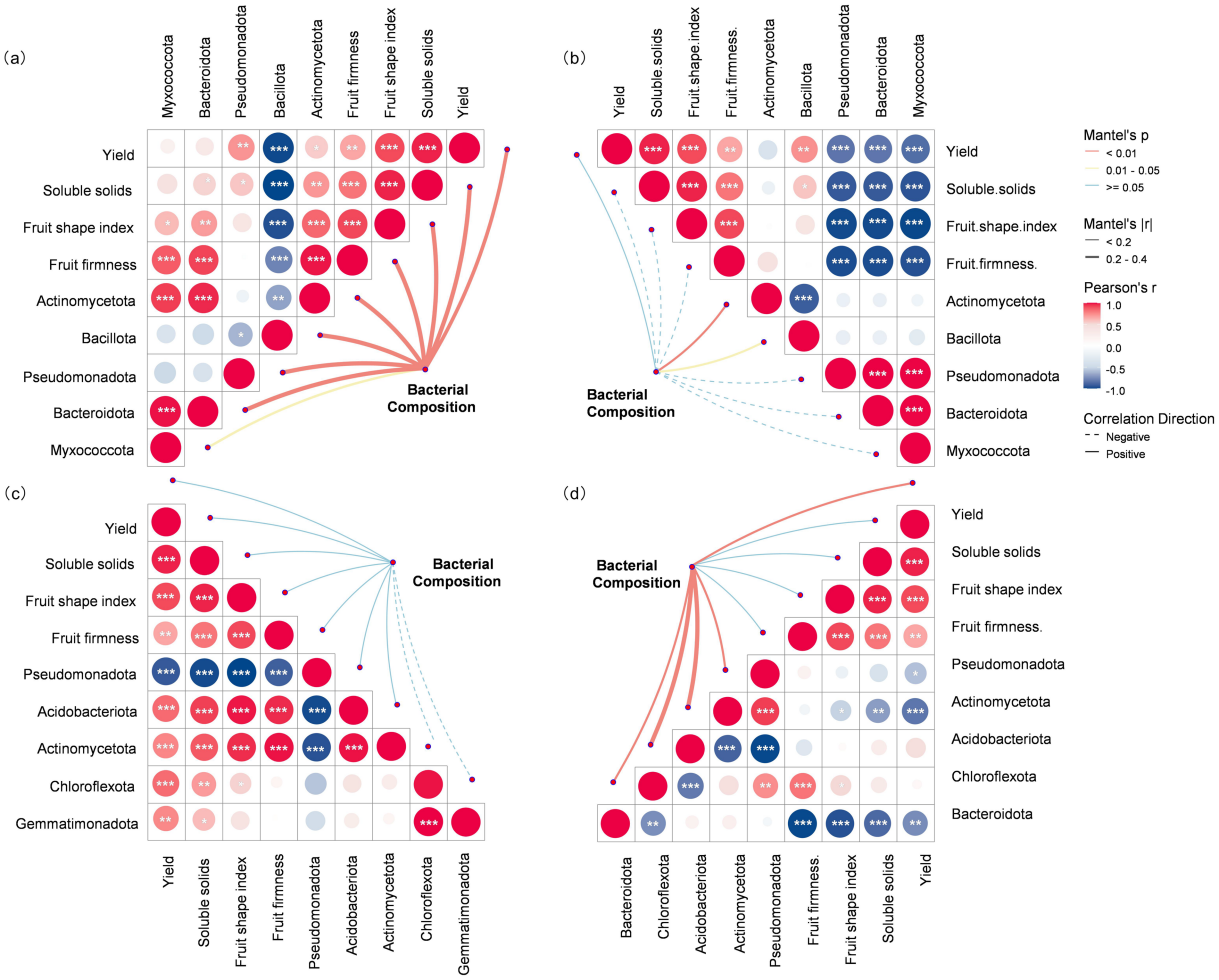
**(a–d)** Relationships between bacterial community composition (based on Bray–Curtis distance) and fruit phenotypic traits or dominant bacterial taxa in the phyllosphere [**(a)** fruit expansion stage; **(b)** maturation stage] and rhizosphere [**(c)** fruit expansion stage; **(d)** maturation stage]. The bacterial community composition was related to each fruit phenotypic trait or dominant bacterial taxon via a Mantel test. Line width corresponds to the Mantel’s *r* statistic (thick = strong correlation, medium = moderate correlation, thin = weak correlation), and line color denotes statistical significance based on permutations (blue = non-significant, yellow = *p* < 0.05, red = highly significant). Pairwise Pearson’s correlations among fruit phenotypic traits and dominant bacterial taxa are shown in the upper triangle, with a color gradient representing the correlation coefficient (solid line = positive correlation, dashed line = negative correlation).

Mantel tests assessed correlations of both pear yield/quality and dominant phyla abundance with the overall bacterial community structure. During the fruit expansion stage, a strong positive correlation was observed between overall phyllosphere community composition and the matrix of yield and quality traits. Separately, at the phylum level, Bacillota abundance showed a significant negative correlation with yield and quality (Figure 4a). During the fruit maturation, the correlation between overall community structure and yield/quality was not significant. At the phylum level, only Actinobacteria showed a weak positive correlation, whereas Pseudomonadota, Bacteroidota, and Myxococcota were significantly negatively correlated with yield and quality (Figure 4b). For the rhizosphere during the fruit expansion stage, the overall bacterial community structure showed no significant correlation with yield or quality metrics. In a separate analysis at the phylum level, the relative abundance of Pseudomonadota was significantly negatively correlated with yield and quality (Figure 4c). During the fruit maturation stage, a strong positive correlation was observed between the overall rhizosphere community composition and yield/quality. Furthermore, the composition was significantly correlated with the combined abundance of the major bacterial phyla (Figure 4d).

## 4 Discussion

### 4.1 Effects of microbial agents on yield and fruit quality

The application of microbial agents significantly improved the yield and fruit quality of ‘Yuluxiang’ pears (Table 1). This aligns with established roles of plant growth-promoting bacteria (PGPB) in enhancing nutrient acquisition, stimulating plant growth, and modifying soil microbial community structure (Liu et al., 2025). The CF treatment yielded the highest production, likely because the soil-applied anti-replant agent suppressed soil-borne pathogens and improved nutrient availability, thereby promoting fruit set and development (Demir et al., 2024). This mechanism is supported by studies showing that microbial inoculants can induce beneficial shifts in resident microbial communities, thereby enhancing plant vigor and productivity (Joshi et al., 2019). Notably, both the CF and CFW treatments significantly increased the soluble solids content of the fruit, indicating enhanced sugar accumulation. This enhancement in the soluble solids content may be associated with the ability of PGPB to facilitate nutrient solubilization and modulate hormone-mediated growth processes, mechanisms documented in studies of plant growth regulation (Qin et al., 2021). Furthermore, microbial agents may influence photosynthate partitioning, thereby directing more carbohydrates to the fruit and increasing the soluble solids content (Lan et al., 2025). The improved fruit shape index under microbial treatments implies a role for PGPB in regulating fruit morphogenesis, likely mediated through hormonal signaling pathways (Zhang et al., 2022). Such effects have been documented in other horticultural crops, where microbial inoculants were found to enhance fruit size and uniformity by modulating endogenous hormone levels and improving photosynthetic efficiency (Wu et al., 2025). These observations underscore the potential of microbial agents as a sustainable tool for optimizing fruit appearance and yield.

### 4.2 Shifts in bacterial community structure in response to microbial treatments

Our results demonstrate that microbial applications significantly restructured bacterial community composition (Figure 1) and altered alpha diversity (Table 2) in both the phyllosphere and rhizosphere, with more pronounced effects in the phyllosphere. This is consistent with Qin et al. (2019), who reported that foliar application of BCAs could substantially modify the phyllosphere microbiota, leading to improved plant health. The reduction in phyllosphere bacterial richness under CFW treatment may reflect a selective enrichment of beneficial taxa such as Actinomycetota, which are known for their pathogen suppression and role in nutrient cycling (García et al., 2025). In the rhizosphere, microbial treatments significantly increased the abundance of Bacillota during the fruit maturation stage (Figure 1d). The relative stability of the rhizosphere community compared to the phyllosphere underscores its resilience but also highlights the potential for long-term modulation through repeated microbial inoculations (Díaz-Rodríguez et al., 2025). This is consistent with previous work showing that microbial inoculants can induce both transient and persistent changes in soil communities, depending on the inoculant and application strategy (Trabelsi and Mhamdi, 2013).

Beyond community composition, microbial applications altered the topological properties of bacterial co-occurrence networks (Figure 3). Both CF and CFW treatments increased the number of nodes and edges across sampling periods, indicating greater network complexity and enhanced microbial interactions. This is consistent with reports linking greater network complexity, particularly in the phyllosphere, to improved plant health and disease suppression (Hilber-Bodmer et al., 2017). The increased density and predominance of positive correlations in these networks suggest a shift toward stronger cooperative interactions and greater functional redundancy. This may enhance the community’s stability and resilience against pathogen invasion (Xing et al., 2024). Moreover, the enrichment of dominant taxa within these networks, particularly Bacillota and Actinomycetota, implies that microbial inoculants not only introduced beneficial strains but also reinforced the ecological roles of indigenous beneficial taxa (García et al., 2025). This principle extends to other beneficial taxa. For example, the genus *Pseudomonas* is well-known for promoting plant growth and suppressing pathogens via secondary metabolites, enzymes, and hormones (Soliman et al., 2023). The integration of such beneficial taxa into more complex and cooperative networks enhances functional redundancy and ecological stability. This restructured environment likely suppresses pathogens through intensified competition for resources and spatial niches (Cui et al., 2025). Principal coordinates analysis further confirmed that microbial treatments drove the formation of distinct bacterial clusters over time (Figure 2). The clear separation of communities in the PCoA plots indicates that inoculants induced significant and persistent shifts in microbiome structure, which aligns with the established capacity of microbial amendments to reshape resident communities (Berg et al., 2021). Collectively, these findings underscore the potential of tailored microbial applications to steer microbiome assembly.

### 4.3 Linking microbial community dynamics to yield and quality

Mantel test analyses revealed significant correlations between the structure of bacterial communities in both the phyllosphere and rhizosphere and the yield and quality of ‘Yuluxiang’ pears (Figure 4). The strong positive correlation between early-stage phyllosphere community structure and fruit yield/quality underscores the critical role of early microbial colonization in shaping development (Mehlferber et al., 2023). This early establishment of a beneficial microbiome may enhance the plant’s physiological state and direct resource allocation toward reproductive growth (Khoiri et al., 2021). This aligns with the understanding that a more complex phyllosphere network can suppress pathogens and improve plant health (Mehlferber et al., 2023). Such complexity fosters a competitive environment that reduces the available ecological niches for pathogens, a mechanism likely contributing to the improved outcomes observed here.

The relationship between the rhizosphere microbial community and plant performance also showed significant dynamics. The shift from a non-significant correlation at the fruit expansion stage to a strong positive correlation at maturation suggests that the influence of microbial inoculants on the rhizosphere microbiome intensifies during critical fruit development. This shift coincided with a significant increase in the abundance of *Bacillota* under the CF and CFW treatments (Figure 4d). *Bacillota species* (e.g., *Bacillus* spp.) are well-documented for their plant growth-promoting and biocontrol traits, including the production of antimicrobial compounds, lytic enzymes, and volatile organic compounds (Marco et al., 2022), as well as their ability to induce systemic resistance in plants (Balthazar et al., 2020). Their enrichment in the rhizosphere likely contributed to the observed improvements in fruit quality, particularly the increase in soluble solids and sugars, by enhancing nutrient availability and uptake (Lan et al., 2025). This aligns perfectly with the findings of, who reported that soil application of PGPB enhances plant growth and physiological performance through rhizosphere activity. This mechanism likely involves improved mineral nutrition and a more robust root system, thereby enhancing photoassimilate production and translocation to developing fruits (Sun and Shahrajabian, 2023). The distinct responses of phyllosphere and rhizosphere communities to microbial applications, evident in our PCoA and Mantel test results, indicate these compartments require targeted management to optimize plant outcomes. The phyllosphere, being an open and harsh environment, may require repeated applications or specific consortia to establish a stable, beneficial community (Hirata et al., 2025). In contrast, the rhizosphere community, while more buffered, exhibits a delayed but significant restructuring in response to inoculation, which has a profound impact on yield (Kui et al., 2021). This intricate interplay between the two compartments, mediated by the plant’s systemic physiology, ultimately determines the final economic output (Hacquard et al., 2022). Therefore, a precision management strategy that separately targets the phyllosphere and rhizosphere microbiomes at key growth stages presents a powerful approach to enhance fruit economic traits and advance sustainable orchard production.

## 5 Conclusion

This study shows that microbial agent application is an effective strategy for enhancing the yield and fruit quality of ‘Yuluxiang’ pears. Among the treatments, soil application of an anti-replant disease agent (CF) led to the greatest yield increase, while the combined soil and foliar microbial treatment (CFW) optimally enhanced fruit quality traits, including soluble solids content, shape, and firmness. These agronomic improvements were strongly associated with a targeted restructuring of the microbiome, characterized by the enrichment of beneficial taxa (e.g., Actinomycetota) and the development of more complex, cooperative microbial networks in both compartments. Notably, the response of the microbial community was compartment-specific: phyllosphere communities were rapidly reshaped, whereas rhizosphere communities exhibited a more gradual but ultimately significant restructuring correlated with final yield. Our results found that comprehensive microbial community management strategies targeting specific compartments can achieve sustainable pear tree production while enhancing fruit quality. Our findings demonstrate that an integrated, compartment-targeted microbiome management strategy can promote sustainable pear production: soil-applied microbial agents alleviate replant disease and enhance yield, while foliar-applied PGPB optimize fruit quality. Together, these applications provide a practical, science-based framework for building more resilient and productive pear cultivation systems.

## Data Availability

The datasets presented in this study can be found in online repositories. The names of the repository/repositories and accession number(s) can be found at: https://www.ncbi.nlm.nih.gov/, SRR36362282.
